# 

**Published:** 1873-05

**Authors:** 


### AMERICAN JOURNAL

OF

### DENTAL SCIENCE,

EDITED BY

F. J. S. GORGAS, A. M., M. D., D. D. S.,

1873-4.

VOL. 7.—THIRD SERIES.

Surgeon Genl's Office
LIBRARY.
53303
Washington, D.C.

BALTIMORE:

SNOWDEN & COWMAN.

LONDON:

TRUBNER & CO., 60 PATERNOSTER ROW.

1874.

### INDEX TO VOLUME VII.---THIRD SERIES.

A Cause of Facial Neuralgia, - - - - - 88
A Dentist Fined for giving Chloroform, - - 381
A Model Hospital, - - - - - - 134
A New Specific for Rheumatism, - - - - 85
A New Method for Producing Local Anæsthesia, - - 230
A New Method for Healing Ulcers, - - - 159
A New Way of Gargling, - - - - - - - 574
A Pre-historic Skull, - - - - - - 381
A Rare and Extraordinary Case, - - - 331
A Remedy for Hæmoptysis, - - - - - 141
A Sure Test as to Whether Life is Extinct or Not, - 92
Action of Cohesive Foil Under the Instrument, - - 53
Action of Nitrous Oxide Gas, - - - - - 330
Adhesion of Soft Palate and Uvula to Posterior Wall of Pharynx, - - - - - - 25
Alleged Death from Ether, - - - - - 451
Aluminum as a Base for Artificial Teeth, - - - 469
Alumni Associations and their Influence for Good, - 29
Amalgam Fillings, - - - - - - 320
Amalgams, Character of - - - - - 519
American Academy of Dental Science, - - 284
American Dental Association, - - 16, 119, 241
American Dental Convention, - - - - 90, 118
Ambler, J. G., D. D. S., - - - - - - 118
Amputation of Part of the Tongue for Carcinomatous Disease, 74
Anæsthesia, The Discovery of - - - - 137
Anodyne Colloid, - - - - - - 48
Application for Chilblains, - - - - - 46
Appropriation of Calcific Elements of Deciduous Teeth for the Permanent Ones, - - - - - 299
Austen, Prof. P. H., M. D., D. D. S., - - - 473
Arthur's Instrument, - - - - - - 45
Artificial Respiration and Transfusion, - - 158
Atropia and Salivation, - - - - - - 380

Bad Tea, - - - - - - 431
Bad Effects of Thumb-sucking, - - - - - 525
Baltimore College of Dental Surgery, - 284, 473, 517
Best Method for Removing Upper Maxillary Bone, - - 32
Bevan, C. F., M. D., - - - - - - 508
Bones from Dissecting Rooms, - - - - 480
BIBLIOGRAPHICAL.
A System of Dental Surgery, - - - 140
A Manual of Dental Mechanics, - - - 140
On Strictures of the Urethra, - - - 141
Practical Histology in Vienna, - - - 382
Microscopical Study of Blood and Epithelium, - 382
Circulars of Information of Bureau of Education, - 382
Mechanism of Ossicles of Hearing and Membrane of the Round Window, - - - 383
Transactions of California State Dental Association, 383
The Sanitarium, - - - - 383
The Herald of Health, - - - - - 383
American Agriculturist, - - - 383
Physicians' Visiting List for 1874, - - - 383
Wood's Household Magazine, - - - 384
Vick's Floral Guide for 1874, - - - 384
Webster's Dictionary, - - - - 427
Dunglison's Medical Dictionary, - - - 571
Transactions of American Dental Association, - 574
Bigelow, Henry, M. D., D. D. S., - - - 451
Birthplace of Modern Dentistry, - - - 373
Bleaching Wax, - - - - - - 335
Blindness Cured by the Extraction of Diseased Teeth, - 424
Brain Stimulants, - - - - - 90
Brunonian or Molecular Movement, - - - 7
Bromide of Potassium in Diseases of Dentition, - 232
California State Dental Association, - - 16, 145
Carbolic Acid as a Local Anæsthetic, - - 47, 370
Carbolic Acid, Fatal Poisoning from - - - 160
Case of Facial Paralysis, - - - - - 366
Causes of Dental Caries, - - - - - - 12
Cavities of Decay in Proximal Surfaces, - - 445
Character of Amalgams, - - - - - - 519
Chisholm, J. J., M. D., - - - - - 32

Children *versus* Crime, - - - - - - 480
Chloroform in Dentistry, - - - - - 528
Chloroform and Morphia, Dangers of - - - 47
Chase, H. S., M. D., D. D. S., - - 125, 310
Chloral for Toothache, - - - - - - 382
Chloral as an application to Fetid Ulcers, - - - 237
Chloral Hydrate, Vehicle for - - - - - 142
Cholera, - - - - - - - 238
Chromic Acid, - - - - - - 46
Chase, Dr. Edward C. - - - - - 337
Chemistry of the Oral Secretions and their Action Upon the Teeth, - - - - - - - 337
Class Address, - - - - - 1, 543
Cooke, A. B., M. D., - - - - - 25
Collis' Method of Operating for Hare Lip, - - - 233
Colorless Tincture of Iodine, - - - - - 89
Cleaning and Smoothing Rubber Plates, - - 351
Cleft Palate, - - - - - - - 574
Corrections, - - - - - - 375
Counter Irritation, The Theory of - - - 371
Convulsions During Dentition, - - - - 475
Counterfeit Diplomas, - - - - 479
Coupein, T. F., D. D S., - - - - - - 80
Cure for Baldness, - - - - - 576
Cutler, S. P., M. D., D. D. S., 7, 56, 120, 150, 223, 226, 266, 349, 533
Chloroform in Tic Douloureux, - - - - - 380
Danger of Morphia and Chloroform, - - - 47
Dangers of Chromic Acid, - - - - - 95
Day, W. H., M. D. - - - - - - 325
Dean, M. S., D. D. S., - - - - - 119, 299
Death During Etherization, - - - - - - 144
Death from Methylene Ether, - - - - 238
Death from Ether in a Dental Office, - - - 402
Dental Associations, 16, 29, 97, 118, 119, 113, 145, 216, 241, 350, 522
Dental Accidents During Anæsthesia - - - 93
Dental Education - - - - - - 329
Dental Physiology, - - - - 125, 273
Dental Pathology, - - - - - 343

Dentist of the Future, The - - - - - 106
Dentistry by the Hour, - - - - - 569
Dentistry, - - - - - - . 570
Decision of Supreme Court of U. S. in Rubber Case, - 36
Detection of Substitution of Carbolic Acid for Creasote, 239
Diagnosis in Practice, - - - - - 102
Diagnosis of Lipomata, - - - - 526
Diarrhœa in Teething, - - - - - 143
Diseases of the Antrum, - - - - 352
Diarrhœa in Teething Children, - - - - 325
Digestive Power of Saliva and Pancreatie Juice During
Infancy, - - - - - 322
Discoverer of Anæsthesia, - - - - 137
Drake, E. D., M. D., - - - - - 327
Edes, R. T., M. D., - - - - - 556
Effect of Nitrous Oxide Gas, - - - - 71
Effects of Alcoholism Upon the Nervous System, - - 77
Effects of Suppression of Perspiration, - 240
Electricity as a Means of Resuscitation, - - - 84
Ergot in Epistaxis, - - - - - 576
Ether, Alleged Death from, - - - 450
Ether Glue, - - - - - - 527
Excision of Left Superior Maxillary and Malar Bones and
One Half of the Nose, - - - - 566
Expansion of the Arch in the Treatment of Irregularity
of the Teeth, - - - - - 521
Facial Paralysis, - - - - - 432
False Teeth, - - - - - - 93
Farmer, John W., M. D., D. D. S,, - - - - 543
Fatal Poisoning from Carbolic Acid, - - - 160
Fletcher, Thos. F. C. S. - - - - - 320
Filling Cavities of Decay in Proximal Surfaces, - 445
Forbes, Isaiah, D. D. S., - - - - - 17
Fragmentary Clippings, - - - - 533
Francis, C. E., D. D. S,, - - - - 561
French, Dr. A. W. - - - - - 111
Fire-Proof Ink, - - - - - 477
Furrowed Enamel in Connection with Syphilitic Diseases, 120
Fundenberg, W. F. M. D., - - - - 271
Furman, Dr, S. M. - - - - - 469

Gelseminum in Odontalgia, - - - - 319
Georgia State Dental Society, - - - - 49
Glycerine Lotion, - - - - - - 95
Growth and Reproduction of Bone, - - - 280
Gunning, Thos. B., M. D., D. D. S. - - - 385, 433
Harris, E. N., D. D. S., - - - - - 350
Hereditary Syphilis, - - - - - - 367
Honor to whom Honor is Due, - - - - 271
Hope for the Bald, - - - - - - 430
Hops as an External Application, - - - - 48
Horvath, A., M. D. - - - - - - 230
Hunter, E. L., D. D. S., - - - - - - 53
Hypertrophy of the Tongue, - - - - - 333
Ice in the Treatment of Facial Neuralgia, - - 334
Incombustible Paper, - - - - - 477
Important Point in the Treatment of Frozen Limbs, - 527
Ingrowing Toe Nails, - - - - - 239
India Rubber, A New Source of - - - - 575
Indiana State Dental Association, - - - 97
Irregularity, - - - - - - - 17
Irritation of Rubber Plates, - - - - 374
Jacobi, Louise, D. D. S., - - - - 460
Kentucky State Dental Association, - - - 97
Labor, - - - - - - - 91
Lancing the Gums, - - - - - - 324
Laws of Transmission of Resemblance from Parents to
Children, - - - - - - 364
Laws of Health, - - - - - - 527
Larynx the Source of Vowel Sounds, - - 382, 433
Lead Poisoning Through Drinking Water, - - - 430
Leeches and How I Manage Them, - - - - 512
Letter from W. H. Morgan, D. D. S., - - - - 473
Lime Water for Stings of Insects, - - - - 528
Lindsey, V. S., M. D. - - - - - - 280
Listening With the Teeth, - - - - - 428
Mason, J. W., M. D., - - - - - - 547
May the Calcific Elements of Deciduous Teeth be Appropriated in the Formation of the Permanent? - 299
Microscopy, - - - - - - - 349
Mental Hygiene, - - - - - - - 523

Mechanical Dentistry, - - - - - - 111
Metallic Enamel, - - - - - - - 425
Methylene Ether as an Anæsthetic, - - - 524
Metivier, M., - - - - - - 566
Mercury in the System, - - - - - 430
Microscopic Terrors, - - - - - - 524
Mississippi Valley Dental Association, - - 522
Missouri State Dental Association, - - - 522
Mixtures of Ether and Chloroform, - - - 67
Model Hospital, - - - - - - - 134
Mode of Operating for Harelip, - - - 431
Nasal Calculus, - - - - - - 379
Needle Swallowed by a Child, - - - 573
Neuralgia of the Trigeminus, - - - - 490
New Method of Treating Ulcers, - - - 83
Neuralgic Pill, - - - - - - - 96
Neuralgia and Sciatica Cured by Turpentine, - - 159
New Method of Producing Local Anæsthesia, - - 230
New Means of Relieving Toothache, - - - 375
New Dental Journals, - - - - - 474
New Local Anæsthetics, - - - - 523
Nervous and Sick Headache, - - - - 526
Nervousness, - - - - - - - 142
Nitrous Oxide Experiments, - - - - 150
Oatmeal, Bone and Muscle, - - - - - 142
Obituary.
Dr. Amos Westcott, - - - - - 287
Dr. Joseph Robinson, - - - - - 288
Dr. Cyrus S. Knapp, - - - - 336
Odor of Orris Root, - - - - - - 144
Old Way and New in Dental Practice, - - 462
Ointment for Neuralgia, - - - - - 335
On Use of Artificial Respiration, - - - 158
On Treatment of Fractures of the Inferior Maxilla, - 81
Oral Surgery, - - - - - - 415
Oxygen, Physiological Effects of - - - - 34
Pathological Dentition, - - - - - - 357
Pepsin, - - - - - - - 556
Persecution of Male Students, - - - - 528
Physiology of the Dental Structures, - - 223, 266
Physiology of the Nervous System, - - - 169

Physiological Effects of Oxygen, - - - 34
Pivot Teeth, - - - - - - - 289
Poisoning Character of Nitrous Oxide, - - - - 334
Porcelain Impression Cups, - - - - - 157
Proceedings of American Dental Association, - - 241
Proceedings of Southern Dental Association, - - - 193
Prof. Heckel on Origin of Man, - - - - - 94
Predisposing Causes of Dental Caries, - - - 12
Professional Excellence, - - - - - 481, 529
Preparation of Cavities, - - - - 561
Prevention of the Loss of Blood, - - - - 429
Reaching Pulp Canals, - - - - - 570
Recent Death in a Dentist's Chair, - - - 552
Remarks on Methylene, - - - - - - 335
Removal of Cystic Tumor from Eyelids, - - - 88
Removal of Foreign Body from Nose, - - - - 375
Replantation of Teeth, - - - - - 286
Restoration of Chloroform, - - - - - 381
Roughening Plugger Points, - - - - 331
Rubber Dam and its Application, - - - - 43
Salivation Produced by Mixing Mercury in the Hands, - 571
Saponin as a Local Anæsthetic, - - - 564
Separating Teeth, - - - - - - 235
Siamese Twins, - - - - - 572
Silver, D. R., M. D. - - - - - - 81
Singular Causes of Death, - - - - - - 47
Southern Dental Association, - - - 16, 89, 133
Southern Dental Association and the Colleges, - 216
Suppression of Perspiration, - - - - 143
Surgery, - - - - - - - - 78
Sugar and Magnesia an Antidote to Arsenic, - - 525
Swallowing Artificial Teeth, - - - - 315
Tea *versus* Brandy, - - - - - - 46
Ten Eyck, J. B., M. D., D. D. S., - - - 1, 12
Test for Impurities in Water, - - - - 432
Testimony in Favor of Dr. Arthur's Method, - - 80
Tempering Steel, - - - - - - - 377
Thackston, W. W. H., M. D., D. D. S., - 481, 529
The Human Body Compared to a Machine, - - - 525
The Dental Rubber Cases—A Novel Law Suit, - - 426

The Blood Cure, - - - - - - - 526
The Legal Value of Teeth, - - - - - 480
The Process of Taking Cold, - - - - - - 288
The Wonders of the Deep, - - - - - - - 96
Tobacco, - - - - - - 547
Treatment of Burns and Scalds, - - - - - 528
Treatment of Chilblains, - - - - - 378
Treatment of Teeth with Dead Pulps, - - - - 384
Treatment of Salivation by Atropia, - - - 332
Treatment of Morbid Dentition, - - - - 328
Treatment of Burns, - - - - - - 336
Treatment of Fracture of Inferior Maxilla, - - - 81
Traumatic Phthisis, - - - - - - 508
Use of Gastric Juice in Treatment of Cancerous Tumors, 432
Use of Sewing Machines, - - - - - - 478
Vehicle for Chloral Hydrate, - - - - - 142
Vienna Mixture, - - - - - - - - 67
Vital Actions which play an Important Part in Dental
Caries, - - - - - - - - 310
Vulcanite Litigation, - - - - - 307, 420, 471
Webb, Marshall, D. D. S., - - - - - - 445
Welchens, Samuel, D. D. S., - - - - - - 462
When to Lance the Gums, - - - - - 324
White, Samuel S., D. D. S., - - - 307, 420, 471
Wilkerson, B. M., M. D., D. D. S., - - - - 175
Women Dentists in Egypt. - - - - - - 528

### *American Dental Association.*

The Committee on Literature of the American Dental Association, would request members of the profession to inform them of any new works which have appeared, or may appear before the next annual meeting of the Association, in order that no work which should properly be considered by the committee, need be overlooked by them.

GEORGE H. CUSHING, Chairman,

550 Michigan Av., Chicago.

E. A. BOGUE, 29 East 20th Street, New York City.

C. N. PIERCE, Philadelphia.

---

### *California State Dental Association.*

The California State Dental Association will commence its fourth annual session in San Francisco, Tuesday, May 20th, 1873, at 10 o'clock A. M., and holding four days. Every member of the profession on the Pacific coast is cordially invited to be present. Clinics daily.

H. J. PLOMTEAUX, *Rec. Sec.*

---

### *Southern Dental Association.*

The fifth annual meeting of this Association will be held in Baltimore, commencing on the last Tuesday in July next.

JAS. F. THOMPSON, M. D., D. D. S.

### TO READERS AND CORRESPONDENTS.

Communications intended for publication, and exchange journals and paper[illegible] should be addressed to the Editor of the American Journal of Denta[illegible] Science, 259 N. Eutaw St., Baltimore, Md. All letters relating to the busine[illegible] of the journal, or containing cash remittances, to be directed to Messrs. Snowde[illegible] & Cowman. Articles for publication should be received by the editor at leas[illegible] three weeks previous to the first day of the month on which the number in whic[illegible] it is designed for them to appear, is issued.

### CONTENTS:

### ORIGINAL COMMUNICATIONS.

☞ The American Journal of Dental Science will contain fifty pages of reading matter in each number, making a volume of not less than

*Six Hundred* pages

EXCLUSIVE OF ADVERTISEMENTS.

THE

### AMERICAN JOURNAL
OF
### DENTAL SCIENCE.

F. J. S. GORGAS, M. D., D. D. S., Editor.

The *Journal* is issued on the first of each month and contains not less than *fifty* pages of reading matter in each number, exclusive of advertisements, making a volume of *six hundred* pages per annum.

In each number will be found Original Communications, Correspondence, Selected Articles, Monthly Summary, Bibliographical Notices and Editorial Matter, and every effort will be exerted to render the *Journal* worthy of the patronage of the Dental profession.

A generous co-operation is respectfully solicited from the members of the profession; and as the *Journal* has now a printing office of its own which materially lessens the cost of its publication, it will be issued at the reduced subscription price of

**$2.50 Per Annum in Advance.**

Communications are respectfully solicited on all subjects pertaining to the practice of dentistry, as well as reports of cases occurring in dental practice.

### RATES OF ADVERTISING.

| | 1 MO. | 2 MO. | 3 MO. | 6 MO. | 12 MO. |
|---|---|---|---|---|---|
| 1 PAGE. | $15 00 | $22 50 | $30 00 | $50 00 | $100 00 |
| ½ " | 10 00 | 15 00 | 20 00 | 30 00 | 50 00 |
| ¼ " | 7 00 | 11 00 | 15 00 | 20 00 | 30 00 |
| ⅛ " | 5 00 | 8 00 | 11 00 | 15 00 | 20 00 |

All original communications designed for publication, works for review, new instruments for notice, exchanges, &c., should be directed to the editor, 259 N. Eutaw St., Baltimore, Md.

All advertisements and subscriptions should be addressed to

SNOWDEN & COWMAN,

*Publishers of the Am. Journal of Dental Science.*

86 W. FAYETTE ST. BET. CHARLES & LIBERTY STS.,

BALTIMORE, Md.

